# CT-Kolonographie: Technik und Indikationen

**DOI:** 10.1007/s00117-023-01153-4

**Published:** 2023-05-30

**Authors:** Thomas Mang, Katharina Lampichler, Martina Scharitzer

**Affiliations:** Universitätsklinik für Radiologie und Nuklearmedizin, Medizinische Universität Wien, Währinger Gürtel 18–20, 1090 Wien, Österreich

**Keywords:** Dickdarmkrebs, Kolorektale Neoplasie, Vorsorge, „Fecal tagging“, Darmdistension, Colon cancer, Colorectal neoplasms, Screening, Fecal tagging, Colonic distension

## Abstract

**Hintergrund:**

Um mit der CT-Kolonographie (CTK) gute Ergebnisse zu erzielen, ist neben der spezifischen radiologischen Expertise eine hochqualitative Durchführung der Untersuchung und eine Indikationsstellung gemäß fachspezifischen Richtlinien erforderlich.

**Ziel der Arbeit:**

Ziel dieser Arbeit ist es, einen Überblick über aktuelle Standards der Untersuchungstechnik sowie über Indikationen und Kontraindikationen der CTK in Anlehnung an rezente Empfehlungen und Richtlinien zu geben.

**Material und Methoden:**

Mittels einer ausführlichen Literaturrecherche wird der aktuelle Wissensstand zur Untersuchungstechnik sowie zu den Einsatzgebieten und den Kontraindikationen zur CTK zusammengefasst.

**Ergebnisse:**

Die CTK ist die radiologische Untersuchung der Wahl zur Detektion kolorektaler Neoplasien. Indikationen sind die unvollständige Koloskopie, Kontraindikationen oder Ablehnung der Koloskopie und die opportunistische Dickdarmkrebsvorsorge. Die Untersuchungstechnik umfasst eine den speziellen Erfordernissen der CTK angepasste Darmvorbereitung einschließlich „fecal tagging“, die Darmdistension, einen Niedrigdosis-CT-Scan in zwei Patientenpositionen sowie eine kombinierte 2D- und 3D-Auswertung.

**Diskussion:**

Die Durchführung der CTK nach aktuellen technischen Standards ist Voraussetzung für hochqualitative und aussagekräftige Untersuchungen und damit auch ein Schlüsselfaktor zur korrekten Diagnosefindung. Als nichtinvasive Untersuchungsoption ermöglicht sie bei vielen Indikationen klinisch relevante Ergebnisse.

## Abstract (en)

**Background:**

Dedicated radiological expertise and a high-quality examination, performed according to current technical standards and for accepted indications, are prerequisite to achieve excellent results with CT colonography (CTC).

**Objectives:**

The aim of this article is to review current standards of the examination technique as well as indications and contraindications for CTC based on recent recommendations and guidelines.

**Materials and methods:**

Based on extensive literature research, current knowledge about the examination technique and the indications and contraindications is summarized.

**Results:**

CTC is the radiological examination of choice for the detection of colorectal neoplasia. Beside incomplete or refused colonoscopy and contraindications to colonoscopy, CTC is also a noninvasive option for opportunistic colorectal cancer screening. The examination technique is based on a CTC-specific patient preparation scheme that includes fecal tagging, colonic distension, low-dose CT scans in two patient positions and a combined 2D and 3D data evaluation.

**Conclusions:**

Performing CTC according to current technical standards is prerequisite for high-quality examinations and is, thus, also a key factor to obtain a correct diagnosis. CTC is a noninvasive examination, capable of providing clinically relevant diagnoses for a wide range of indications.

## Hintergrund

**Figure 1:**
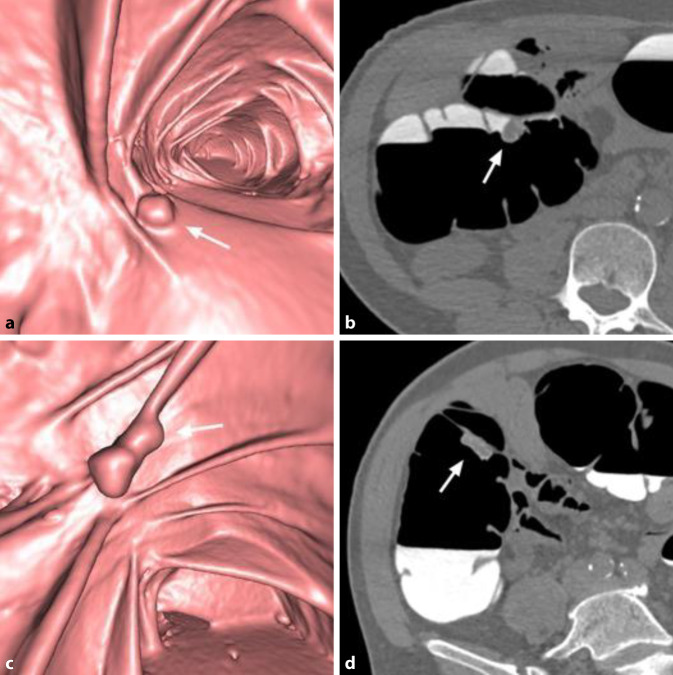

Die CT-Kolonographie (CTK) oder auch virtuelle Dickdarmspiegelung ist eine leistungsfähige radiologische Untersuchung zur nichtinvasiven Dickdarmdiagnostik. Sie ist der beste radiologische Test zur Detektion kolorektaler Neoplasien und dem Kolonkontrasteinlauf deutlich überlegen [1, 2]. Die CTK weist eine gleich hohe Detektionsrate für kolorektale Karzinome (KRK) und große Polypen wie die optische Koloskopie auf, und zwar sowohl bei PatientInnen mit Symptomen als auch bei Untersuchungen zur Dickdarmkrebsvorsorge (Abb. 1; [3–5]).

Um derart gute Ergebnisse auch in der klinischen Routine erzielen zu können, ist neben der spezifischen radiologischen Expertise auch die hochqualitative Durchführung der Untersuchung gemäß fachspezifischen Richtlinien erforderlich. Eine unzureichende Untersuchungsqualität schränkt die Beurteilbarkeit und Aussagekraft der Untersuchung ein und macht die radiologische Auswertung komplizierter und zeitintensiver.

Die klinischen Indikationen zur CTK unterliegen ständigen Anpassungen. Sie ergeben sich aus der methodischen Weiterentwicklung, der Berücksichtigung neuer Studienerkenntnisse und aus sich ändernden Anforderungen der zuweisenden Ärztinnen und Ärzte [6].

In der vorliegenden Übersichtsarbeit werden die Untersuchungstechnik sowie Indikationen und Kontraindikationen zur CTK in Anlehnung an aktuelle Empfehlungen und Richtlinien zusammengefasst.

## Untersuchungstechnik

Die Untersuchungstechnik der CTK umfasst Darmvorbereitung, Darmdistension, CT-Scan und Auswertung. Sie wurde in den Richtlinien verschiedener Fachgesellschaften zusammengefasst (Tab. 1; [7–9]).

**Table 1:** 

*Darmvorbereitung*	
Diät	24 h reine Flüssigkeitsdiät Optional 24–72 h faserarme Diät
Darmreinigung	Laxativum zur vollständigen Darmreinigung am Nachmittag vor der Untersuchung (z. B. 2 l Polyethylenglykol-Lösung)
„Fecal tagging“	50 ml jodhaltiges Kontrastmittel oral am Abend vor der Untersuchung
*Darmdistension*	
Darmrohr	Dünne und flexible Darmrohre mit Katheterballon
Spasmolytika	Hyoscin-N-Buthylbromid i.v. (Buscopan®; 20 mg)
Distensionsmethode	Automatisch mit CO_2_ Optional: Raumluft manuell mittels Handpumpe
Patientenlagerung	Bauch- und Rückenlage Optional Seitenlage
*CT-Scan*	
MDCT-Scanner	≥ 16 Zeilen
Topogramm	Obligat vor jedem Scan zur Kontrolle der Distension
Scanrichtung	Kraniokaudal
Effektive Schichtdicke und Rekonstruktionsintervall	SD ≤ 1,25 mm RI ≤ 0,7
Dosisparameter	Niedrigdosisprotokolle (Bauch- und Rückenlage nativ, ≤ 50 mAs) Normaldosis bei i.v.-Kontrastmittel (Bauchlage nativ, ≤ 50 mAs; Rückenlage mit KM, ≥ 100 mAs) Dosismodulationsalgorithmen, iterative Rekonstruktion
Intravenöse (i.v.) Kontrastmittel Applikation	Zur Evaluation extrakolischer Strukturen Kontraindiziert bei Vorsorgeuntersuchungen

*MDCT* Multidetektor-Computertomographie

### Darmvorbereitung

Um das Kolon endoluminal beurteilen zu können, ist eine spezifische, für die speziellen Anforderungen der CTK optimierte Darmvorbereitung notwendig [10]. Darmvorbereitungsprotokolle umfassen eine spezielle Diät, die Darmreinigung mittels Laxanzien sowie die orale Gabe von Röntgenkontrastmittel. Sie sollten unkompliziert gestaltet und für PatientInnen leicht zu befolgen sein. Darüber hinaus sollte die Untersuchung für PatientInnen wenig belastend sein, ein Umstand, der für die Akzeptanz der Methode wichtig ist [11].

#### Diät

Mittels einer Diät wird das Stuhlvolumen sowie auch die Heterogenität des Darminhalts reduziert. Unterschieden wird zwischen einer reinen Flüssigkeitsdiät und einer faserarmen Diät. Die reine Flüssigkeitsdiät beginnt 12–24 h vor dem Untersuchungstermin. In dieser Zeit sollen PatientInnen keine feste Nahrung zu sich nehmen. Erlaubt sind klare Flüssigkeiten, Energy-Drinks und Trinkmahlzeiten. Eine reine Flüssigkeitsdiät ist zwar leicht verständlich und führt zu weniger residualem Darminhalt, ist für PatientInnen allerdings auch belastend.

**Figure 2:**
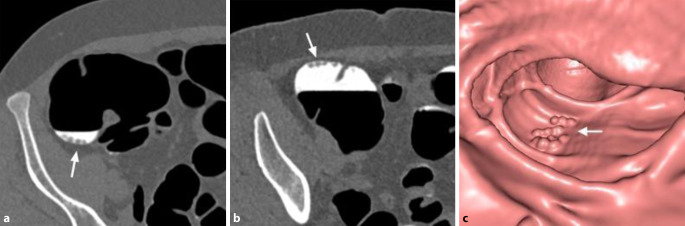

Alternativ wird in vielen europäischen Zentren auch eine faserarme Diät durchgeführt. Dabei werden 24–72 h vor der Untersuchung Nahrungsmittel vermieden, bei denen unverdauliche Reste im Darm verbleiben, wie beispielsweise Gemüse, Früchte, Cerealien und Milchprodukte (Abb. 2). Empfohlen werden hingegen leicht verdauliche Nahrungsmittel wie gekochter Fisch, geschälte Kartoffeln oder Eier und Pasta mit Butter. Faserarme Diäten sind für PatientInnen weniger belastend. Sie sind in der Durchführung allerdings komplizierter und führen zu größeren Mengen residualen Darminhalts. Am Tag der Untersuchung sollte grundsätzlich nur noch klare Flüssigkeit eingenommen werden.

Es ist wichtig, dass PatientInnen während der gesamten Darmvorbereitung ausreichend Flüssigkeit zu sich nehmen.

#### Laxanzien

Residualer Stuhl kann nicht nur relevante Pathologien verdecken, sondern auch simulieren. Er erschwert so die endoluminale Beurteilbarkeit der Untersuchung erheblich (Abb. 2). Um den Darm vollständig von Stuhl zu reinigen, nehmen die PatientInnen am Tag vor der Untersuchung Laxanzien ein. Die laxative Darmentleerung sollte bei der CTK auf maximal 24 h begrenzt werden [7]. Grundsätzlich eignet sich für die CTK jedes Abführmittel, das eine vollständige Darmvorbereitung gewährleistet. In Europa werden für die CTK meistens Polyethylenglykol-Lösungen (Magrocol) oder Sodium Picosulfat verwendet.

Magrocol bindet residuale Flüssigkeit im Darm und verhindert deren Resorption. Es ist inert und wird nicht vom Körper aufgenommen. Magrocol führt zu keiner aktiven Dehydratation und eignet sich deshalb auch für PatientInnen mit Nieren- oder Herzerkrankungen. Nachteilig sind größere Trinkvolumina vieler verfügbarer Präparate sowie damit einhergehend größere Mengen residualer Flüssigkeit. Aus diesem Grund werden anstelle der herkömmlichen 4‑l-Lösungen Präparate mit einem reduzierten Trinkvolumen von 2 l bevorzugt (z. B. Moviprep, Norgine Deutschland).

Sodium Piccolsulfat (z. B. Citrafleet, Recordati Pharma GmbH, Italien) ist ein Abführmittel, das die Darmmotilität stimuliert und so zu einer reduzierten Flüssigkeitsresorption führt. Im Gegensatz zu Magrocol sind die Trinkmengen geringer. Bei PatientInnen mit eingeschränkter Nierenfunktion sollte aufgrund des Risikos von Elektrolytstörungen und Dehydratation auf isoosmolare Polyethylenglykol-Lösungen ausgewichen werden.

Laxanzien werden üblicherweise im Verlauf des Nachmittags vor der Untersuchung eingenommen. Begleitend sollen PatientInnen ausreichend Flüssigkeit zu sich nehmen.

#### „Fecal tagging“

Selbst nach einer Diät und der Gabe eines Abführmittels verbleiben meist residuale Stuhl- und Flüssigkeitsreste im Darm. Aufgrund ähnlicher Dichtewerte können weichteildichte Dickdarmläsionen innerhalb residualer Flüssigkeit nicht erkannt werden. Solide Stuhlreste können überdies auch Kolonpolypen simulieren. Das kann zu falsch-negativen oder falsch-positiven Diagnosen führen.

**Figure 3:**
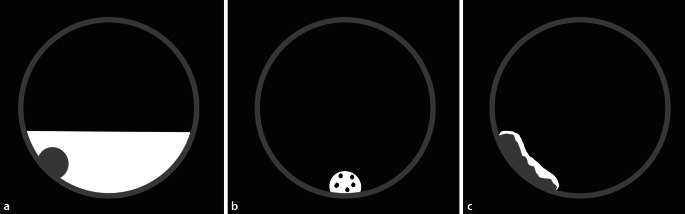

Durch die orale Gabe von jod- oder bariumhaltigen Röntgenkontrastmitteln am Abend vor der Untersuchung kommt es zur hyperdensen Kontrastierung von residualem Darminhalt [12]. Dies ist ein erwünschter Effekt, der als „fecal tagging“ bezeichnet wird. Mittels „fecal tagging“ können weichteildichte Läsionen innerhalb der hyperdensen residualen Flüssigkeit erkannt und hyperdense, polypoide mit Kontrastmittel (KM) vermischte Stuhlreste leicht identifiziert werden (Abb. 3). „Fecal tagging“ steigert sowohl die Sensitivität als auch die Spezifität der CTK. Es ist ein wesentlicher Grund für das gute Abschneiden der CTK in rezenteren Studien [13–15]. Diese Technik hat sich in der klinischen Praxis als so vorteilhaft erwiesen, dass Untersuchungen ohne „fecal tagging“ vermieden werden sollten [7].

Am häufigsten werden ionische hyperosmolare, jodhaltige Kontrastmittel, wie beispielsweise Gastrografin® (Bayer Vital GmbH, Deutschland), eingesetzt. Sie haben zudem einen starken laxativen Effekt. Gebräuchliche Dosierungen bei der CTK liegen zwischen 50 und 100 ml, wobei aus unserer Erfahrung eine Einzeldosis von 50 ml Gastrografin®, am Abend vor der Untersuchung verabreicht, für ein qualitativ hochwertiges, homogenes „fecal tagging“ ausreichend ist.

Jodhaltige Kontrastmittel können zu Dehydrierung und zu Elektrolytverschiebungen führen. Zudem besteht das theoretische Risiko einer anaphylaktischen Reaktion, das bei oraler KM-Gabe allerdings verschwindend gering ist [16].

Bariumsuspensionen werden alternativ zu jodhaltigen KM bei PatientInnen mit bekannter Kontrastmittelunverträglichkeit empfohlen. Sie sind inert, haben keine Nebenwirkungen und sind nicht abführend. Die Qualität des Taggings ist aufgrund von Sedimentation und Ausbildung eines muralen Kontrastmittelbelags weniger gut. Eine nachfolgende Koloskopie kann durch Bariumreste erschwert werden. Bariumsuspensionen werden für die CTK nur noch selten verwendet. Zur Anwendung kommen 225 ml 4,9 %ige Bariumsuspensionen (z. B. E‑Z-CAT, Bracco, Italien), die am Abend vor der Untersuchung getrunken werden.

Die in der amerikanischen Literatur oft als vorteilhaft beschriebene Kombination von jodhaltigem Kontrastmittel mit Bariumsuspensionen wird in Europa kaum angewendet [17]. Die Verabreichung eines weiteren Präparates macht die Vorbereitung komplizierter, was auch die Patientencompliance reduzieren kann [7].

##### „Contrast coating“.

Ein erst rezent beschriebener Nebeneffekt von „fecal tagging“ ist die Adhärenz von Kontrastmittel an der Oberfläche von Kolonläsionen, „contrast coating“ genannt [18]. Das ist bei der Detektion flacher und serratierter Läsionen hilfreich, da Läsionen aufgrund der Kontrastmittelschicht auf 3D-Ansichten größer erscheinen und auf 2D-Bildern besser erkennbar sind (Abb. 3 und 4).

**Figure 4:**
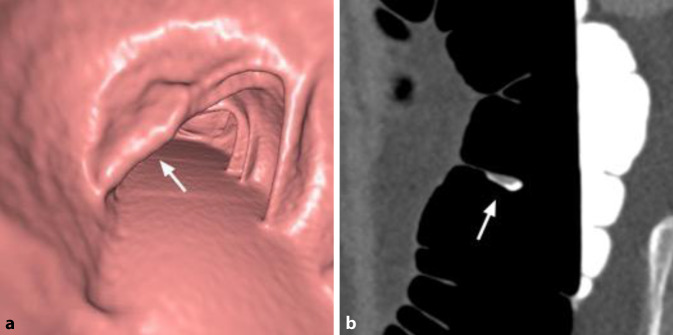

#### Reduzierte oder *laxanzienfreie* Darmvorbereitung

Neben der vollständigen Darmvorbereitung werden immer öfter sog. reduzierte Vorbereitungen eingesetzt, bei denen die Menge des Abführmittels verringert wird, und zwar beispielsweise von 200 g auf 130 g Magrocol [11].

Bei laxanzienfreien Vorbereitungen wird auf die Gabe eines dedizierten Abführmittels vollständig verzichtet. Sie basieren nur auf einer Diät und der alleinigen oralen Gabe eines jodhaltigen Kontrastmittels, das allerdings wesentlich höher dosiert wird (150 ml; [19]). Der laxative Effekt hyperosmolarer, jodhaltiger Kontrastmittel führt jedoch ebenfalls zu einer ausreichenden Darmreinigung, weshalb der Begriff „laxanzienfrei“ streng genommen nicht zutrifft. Laxanzienfreie Vorbereitungsprotokolle sind für PatientInnen weniger belastend und einfacher zu befolgen und sie erhöhen die Akzeptanz der Untersuchung. Auch gelten sie als schonende Alternative für ältere und gebrechliche PatientInnen, die eine komplette Vorbereitung weniger gut tolerieren, und bei denen ein Malignitätsausschluss und nicht die Polypendetektion im Vordergrund steht [7]. Durch die größeren Mengen residualen Darminhalts können sie aber die Aussagekraft der Untersuchung hinsichtlich Kolonpolypen einschränken.

#### Patienteninformationsblatt

Anweisungen zur Diät sowie auch zur Einnahme von Laxanzien und oralem Kontrastmittel müssen in einem Patenteninformationsbogen einfach nachvollziehbar und in leicht verständlicher Form illustriert werden [7].

### Darmdistension

Um den Dickdarm endoluminal einsehen zu können, ist die vollständige Distension des Kolons mittels Luft oder CO_2_ notwendig. In kollabierten Segmenten können kolorektale Neoplasien hingegen nicht ausgeschlossen werden.

Die Darmdistension erfolgt über dünne und flexible Darmrohre, die in Seitenlage rektal eingebracht werden. Ein kleinvolumiger aufblasbarer Ballon an der Katheterspitze verhindert die Dislokation des Katheters und unterstützt auch PatientInnen, das Gas im Darm zu halten. Der Katheterballon sollte kurz vor der Akquisition des zweiten CT-Scans entlastet werden, um distale rektale Läsionen nicht zu verdecken.

Spasmolytika sollen das Ausmaß der Distension verbessern und Spasmen und Beschwerden verringern. Ihr Einsatz wird von der European Society of Gastrointestinal and Abdominal Radiology (ESGAR) empfohlen. Hyoscin-N-Buthylbromid (Buscopan®, Sanofi-Aventis Deutschland GmbH) ist hier das bevorzugte Produkt [7]. Üblicherweise werden 20 mg intravenös vor Beginn der Darmdistension appliziert [9]. Glukagon hat bei der CTK einen geringeren Effekt auf die Darmdistension und wird deshalb von einigen Fachgesellschaften nicht empfohlen [8, 20].

Die Distension kann entweder manuell mit Raumluft über einen Handballon oder automatisch mit CO_2_ über einen Insufflator erfolgen [7]. CO_2_-Insufflatoren steuern sowohl die Gaszufuhr als auch den intraluminalen Druck und verfügen über Sicherheitseinrichtungen, die Druckspitzen reduzieren und Perforationen verhindern sollen. Sie vereinfachen die Gasinsufflation, erzielen eine bessere Distension als die manuelle Luftinsufflation und werden von der ESGAR bevorzugt empfohlen [7]. CO_2_ wird überdies sehr schnell über die Darmwand absorbiert und über die Lunge abgeatmet, was sich nach der Untersuchung positiv auf den Patientenkomfort auswirkt. Die manuelle Luftinsufflation ist allerdings eine akzeptierte und kosteneffiziente Alternative.

Die für die Distension notwendige Gasmenge ist individuell verschieden und hängt von der Länge und dem Volumen des Dickdarms ab. Übliche Gasvolumina liegen bei 3–4 l bzw. bei 40–50 Pumpzyklen mit dem Handballon. Angaben zu standardisierten Gasmengen oder Pumpzyklen als alleinige Parameter für eine vollständige Darmdistension sind allerdings nicht zweckdienlich. Viel effektiver ist es, auf die Toleranz der PatientInnen zu achten. Gibt die Patientin/der Patient ein stärkeres Blähungsgefühl an und toleriert keine weitere Gasinsufflation, ist die Distension in den allermeisten Fällen ausreichend. Die Kommunikation mit den PatientInnen während der Darmdistension ist daher sehr wichtig.

Der Dickdarm gilt als ausreichend distendiert, wenn alle Kolonsegmente in zumindest einer, jedoch idealerweise in beiden Scanpositionen komplett entfaltet sind [7]. Die vollständige Distension aller Segmente wird vor jedem CT-Scan mittels Topogramm kontrolliert. Sind einzelne Segmente am Topogramm nicht ausreichend distendiert, muss weiter Gas insuffliert und ein erneutes Topogramm zur Kontrolle durchgeführt werden.

Automatische Distensionsgeräte erhalten während der Untersuchung den zuvor eingestellten intraluminalen Druck. Bei manueller Insufflation wird üblicherweise zwischen beiden CT-Scans nach Umlagerung des Patienten weiteres Gas bis zur Toleranzgrenze insuffliert.

### CT-Scans in zwei Positionen

Der CT-Scan erfolgt in zwei verschiedenen Scanpositionen („dual positioning“).

Standardmäßig wird in Rücken- und Bauchlage untersucht. Durch die Umlagerung kommt es zu einer Umverteilung von Distensionsgas und von residualer Flüssigkeit sowie auch von Stuhlresten im Kolon. Dadurch können mehr Darmwandabschnitte bzw. Segmente beurteilt werden als in einer einzelnen Scanposition [21]. Darüber hinaus ist die Umpositionierung für die Interpretation intraluminaler Befunde diagnostisch hilfreich, da Kolonläsionen an der Dickdarmwand haften und somit lagestabil sind, während sich Stuhlreste naturgemäß der Schwerkraft entsprechend verlagern.

Ist eine Lagerung des Patienten in Bauchlage nicht möglich, kann stattdessen auch ein CT-Scan in Seitenlage durchgeführt werden. Bei nativen Untersuchungen ohne intravenöse KM-Applikation besteht keine Empfehlung hinsichtlich der Reihenfolge der Scanpositionen.

Eine Voraussetzung für die CTK ist der Einsatz von CT-Geräten, die eine Dünnschichtuntersuchung von Abdomen und Becken in einer kurzen Atemanhaltephase (< 25 s) durchführen können [7, 8]. Hierbei handelt es sich um Multidetektor-CT-Geräte (MDCT) mit zumindest 16 oder mehr Detektorzeilen [10]. Zur Reduktion von Atemartefakten sollten die CT-Scans in kraniokaudaler Scanrichtung erfolgen.

Allgemein wird eine effektive Schichtdicke von 1 mm (≤ 1,25 mm) für die CTK als optimal angesehen [8]. Die Akquisition solcher isotropen Datensätze ist eine grundlegende Voraussetzung für nachfolgende Rekonstruktionen multiplanarer 2D- und endoluminaler 3D-Ansichten. Zur Erzielung einer guten Bildqualität werden überlappende Schichten rekonstruiert (20–30 % Überlappung; [7]).

Vorsorgeuntersuchungen werden routinemäßig mit Niedrigdosisprotokollen ohne intravenöse KM-Applikation durchgeführt [7, 8]. Es wird eine Röhrenspannung von 120 kV und ein Röhrenstrom-Zeit-Produkt von ≤ 50 mAs in Rücken- und Bauchlage empfohlen [8, 9]. Durch zusätzliche Verwendung von Dosismodulationsalgorithmen und iterativer Rekonstruktion lassen sich problemlos effektive Dosiswerte von unter 3 mSv erreichen, ohne wesentliche Einschränkungen der Bildqualität [8, 22].

### Intravenöse Kontrastmittelapplikation

Die intravenöse KM-Applikation ist für die Dickdarmevaluation wegen des großen Kontrasts zwischen Weichteilgewebe, gasdistendiertem Dickdarmlumen und kontrastmittelmarkiertem residualem Darminhalt nicht notwendig. Für Vorsorgeuntersuchungen von asymptomatischen Personen ist sie nicht indiziert. Die Beurteilbarkeit extrakolischer Strukturen ist bei solchen nativen Niedrigdosisuntersuchungen allerdings eingeschränkt.

Bei klinischen Fragestellungen, die auch die Darstellung der extrakolischen Organe erfordern, ist die intravenöse KM-Applikation hingegen indiziert. Dabei erfolgt der erste Scan in Bauchlage nativ in Niedrigdosistechnik. Der zweite Scan wird in Rückenlage kontrastmittelverstärkt und in Normaldosis (≥ 100 mAs) in einer portalvenösen Phase durchgeführt [7, 9].

#### Nach der Datenakquisition.

**Figure 5:**
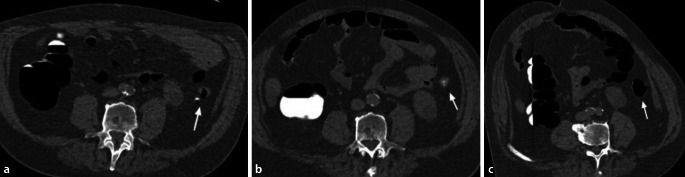

Bevor die Patientin/der Patient den CT-Tisch verlässt, sollte die Untersuchung hinsichtlich ihrer Vollständigkeit und Qualität kontrolliert werden. Lässt sich ein Segment in beiden CT-Scans nicht ausreichend distendieren, sollte in der gleichen Sitzung ein dritter CT-Scan in Seitenlage erfolgen (Abb. 5). Die Darmdistension erfordert spezifische Expertise und sollte durch qualifiziertes Personal erfolgen [7].

#### Probleme und Lösungsstrategien.

Ist die Darmvorbereitung insuffizient, sodass aufgrund großer Mengen von residualem Stuhl fortgeschrittene Neoplasien nicht ausgeschlossen werden können, sollte die Untersuchung abgebrochen und nach erneuter Darmvorbereitung an einem anderen Tag wiederholt werden.

Bei fehlendem „fecal tagging“ und ansonsten zufriedenstellender Darmvorbereitung kann vor Ort 50 ml Gastrografin verabreicht werden. Nach etwa 3 h Wartezeit hat das Kontrastmittel den Gastrointestinaltrakt passiert, und die Untersuchung kann am selben Tag ohne erneute Darmvorbereitung durchgeführt werden.

Diese Vorgehensweise eignet sich auch dann, wenn eine CTK unmittelbar nach einer unvollständigen Koloskopie durchgeführt werden soll. Ein Gespräch mit der zuweisenden Endoskopikerin/dem zuweisenden Endoskopiker zur spezifischen Indikationsstellung und zum Ausschluss assoziierter Kontraindikationen zur CTK (z. B. tiefe Biopsie, Polypektomie, akute Kolitis) ist obligat.

### Bildanalyse

Die Bildauswertung einer CTK erfolgt auf Computerworkstations mit spezifischer Auswertesoftware. Diese verfügen über endoluminale 3D-Ansichten, diverse 3D-Tools sowie multiplanare 2D-Ansichten und erlauben eine simultane Darstellung der Bilddaten von Bauch- und Rückenlage [23].

Die Auswertung von CTK-Datensätzen erfolgt routinemäßig mit einer Kombination von 3D- und 2D-Methoden. Diese kombinierte Auswertung ist der alleinigen Auswertung von 2D-Bildern überlegen [24]. Wahlweise kann primär 2D oder 3D, unterstützt von den jeweils korrespondierenden anderen Ansichten, ausgewertet werden. Welche Methode gewählt wird, hängt u. a. auch von der Erfahrung und der Präferenz der RadiologInnen und der Qualität der Untersuchung ab.

Die primäre 2D-Auswertung erlaubt sowohl eine komplette Darstellung der Darmwand mittels weiter Fenstereinstellungen (sog. Kolonfenster; Weite: 1500 HU und Center: −150 HU) als auch eine Differenzierung der Darmwandstrukturen und Läsionen mittels enger Fenstereinstellungen (Abdomenfenster; Weite: 400 HU und Center: 40 HU). 3D-Ansichten werden unterstützend zur Befundanalyse eingesetzt.

Bei Verwendung endoluminaler 3D-Ansichten zur primären Bildanalyse muss die Auswertung bidirektional erfolgen, um Areale, die bei unidirektionaler Untersuchung dem virtuellen Blickwinkel entgehen, einsehen zu können [25]. Suspekte Befunde werden mithilfe von 2D-Ansichten weiter spezifiziert. Primäre 3D-Auswertungen erleichtern zwar die Polypenerkennung, sind aber zeitaufwendiger als primäre 2D-Auswertungen.

Die Befundkriterien der CTK wurden bereits an anderer Stelle ausführlich beschrieben [26].

Bei Verfügbarkeit eines computerassistierten Detektionssystems (CAD) kann dieses in einem Zweit-Leser-Schema, also erst nach einer radiologischen Auswertung des Patientendatensatzes, unterstützend eingesetzt werden [27, 28].

Die vollständige Evaluation einer CTK erfordert die komplette radiologische Auswertung der CT-Scans beider Patientenpositionen. Das dient nicht nur der gesamthaften Erfassung aller Dickdarmsegmente, sondern auch der Beurteilung potenzieller Läsionen. Die zusätzliche Begutachtung extrakolischer Strukturen und Organe ist obligat [7, 9].

## Indikationen und Kontraindikationen

Die folgende Darstellung der Indikationen und Kontraindikationen orientiert sich größtenteils an den aktualisierten konsensuellen Empfehlungen der European Society of Gastrointestinal Endoscopy (ESGE) und der ESGAR aus 2020 [1] unter Berücksichtigung der Empfehlungen anderer Fachgesellschaften [8, 9].

### Radiologische Abklärung kolorektaler Neoplasien

Die CTK ist das beste radiologische Verfahren und auch die beste nichtinvasive Untersuchung zur Abklärung kolorektaler Neoplasien. Bei der klinischen Fragestellung nach kolorektalen Neoplasien wird sie daher als radiologische Untersuchung der Wahl empfohlen [1].

Gleichzeitig besteht eine klare Empfehlung gegen den Kolonkontrasteinlauf. Diese, auch Irrigoskopie genannte, konventionelle Durchleuchtungsuntersuchung ist mittlerweile methodisch obsolet und der CTK aufgrund der signifikant geringeren Sensitivität weit unterlegen [2].

### Spezifische Einsatzgebiete

Bei den Indikationen zur CTK wird zwischen der Abklärung von Symptomen oder spezifischen klinischen Fragestellungen und der Vorsorgeuntersuchung an asymptomatischen PatientInnen mit durchschnittlichem Dickdarmkrebsrisiko unterschieden (Tab. 2).

**Table 2:** 

Radiologische Abklärung kolorektaler Neoplasien	*CTK ist die radiologische Untersuchungsmethode der Wahl* *Empfehlung gegen Kolonkontrasteinlauf*
Diagnostische Fragestellungen/symptomatische PatientInnen	*Inkomplette oder abgebrochene Koloskopie* *Koloskopie kontraindiziert oder nicht möglich*
	Bei KRK-Alarmsymptomen Bei FIT-positiven PatientInnen Postoperativ zur Nachsorge bei kolorektalem Karzinom (kurativ) Verlaufskontrolle nach Polypektomie von Hochrisikopolypen
	*Symptomatische PatientInnen ohne KRK-Alarmsymptome (optional zur Koloskopie)*
Screening	*Opportunistische Dickdarmkrebsvorsorge (optional zur Koloskopie)* *Keine Empfehlung in einem organisierten populationsbasierten Vorsorgeprogramm basierend auf FIT*

*FIT* fäkaler immunochemischer Test, *KRK* kolorektales Karzinom

#### Diagnostische Fragestellungen/symptomatische PatientInnen

Wesentliche diagnostische Indikationen zur CTK sind die unvollständige oder nicht erfolgreich durchführbare Koloskopie sowie auch Kontraindikationen zur Koloskopie.

Eine alternative Untersuchung zur vollständigen Dickdarmabklärung ist dabei besonders für Patientengruppen mit erhöhtem Neoplasierisiko wichtig. Dies sind PatientInnen mit Alarmsymptomen für ein kolorektales Karzinom sowie auch solche mit positivem Test auf okkultes Blut im Stuhl im Rahmen von organisierten Bevölkerungs-Screening-Programmen [1]. Des Weiteren gehören auch PatientInnen zur Nachsorge nach kurativer Resektion eines KRK sowie zur Verlaufskontrolle nach Polypektomie von Hochrisikopolypen dazu, da bei ihnen das Risiko für zukünftige kolorektale Neoplasien erhöht ist [1].

##### Unvollständige Koloskopie.

**Figure 6:**
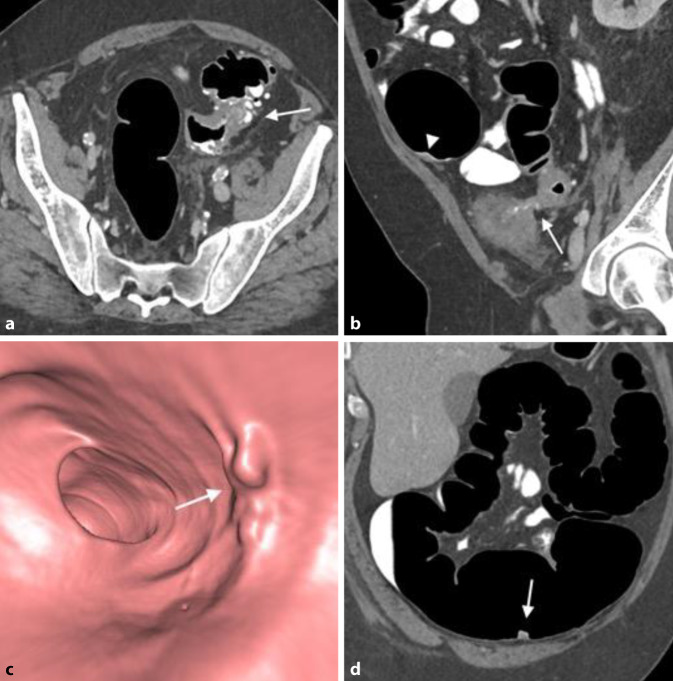

Die CTK erlaubt bei fast allen PatientInnen eine vollständige Darstellung aller Darmsegmente. Sie kann damit auch Erkrankungen in endoskopisch nicht einsehbaren Darmabschnitten aufzeigen [29]. Sie wird besonders dann empfohlen, wenn stenosierende oder morphologische Gegebenheiten für die unvollständige Endoskopie verantwortlich sind und deshalb eine Wiederholung der Koloskopie nicht erfolgversprechend ist. Ursächlich können beispielsweise ein stenosierender Tumor, eine komplizierte Divertikulose, postoperative Adhäsionen oder postentzündliche Strikturen, aber auch eine komplexe anatomische Topografie des Kolons sein (Abb. 6). Die Durchführung der CTK am selben Tag im Zuge der gleichen Vorbereitung ist anzustreben, sofern keine Polypektomie oder eine mukosale Resektion stattgefunden hat.

Bei nichtstenosierenden Befunden, wie beispielsweise einer Kolitis, wird hingegen eine Wiederholung der Koloskopie empfohlen.

##### Kontraindikation zur Koloskopie oder Ablehnung der Untersuchung.

Die CTK ist nichtinvasiv und für PatientInnen weniger belastend als eine Dickdarmspiegelung [30]. Sie eignet sich deshalb gut als Alternativuntersuchung für Personen, bei denen die Koloskopie kontraindiziert oder nicht möglich ist, aber auch für Personen, die endoskopische Untersuchungen ablehnen.

##### Symptomatische PatientInnen ohne KRK-Alarmsymptome.

Bei symptomatischen PatientInnen ohne spezifische Alarmsymptome für ein kolorektales Karzinom ist die CTK eine gleichwertige Alternative zur Koloskopie. Es kann sowohl eine Koloskopie als auch eine CTK durchgeführt werden. Eine vorangegangene frustrane Koloskopie ist zur Indikationsstellung nicht notwendig [1].

#### Empfehlungen zur Dickdarmkrebsvorsorge

Die Empfehlungen zum Einsatz der CTK in der Dickdarmkrebsvorsorge sind von der Art des Vorsorgeprogramms abhängig. Dabei wird zwischen opportunistischer und organisierter populationsbasierter Vorsorge unterschieden.

**Figure 7:**
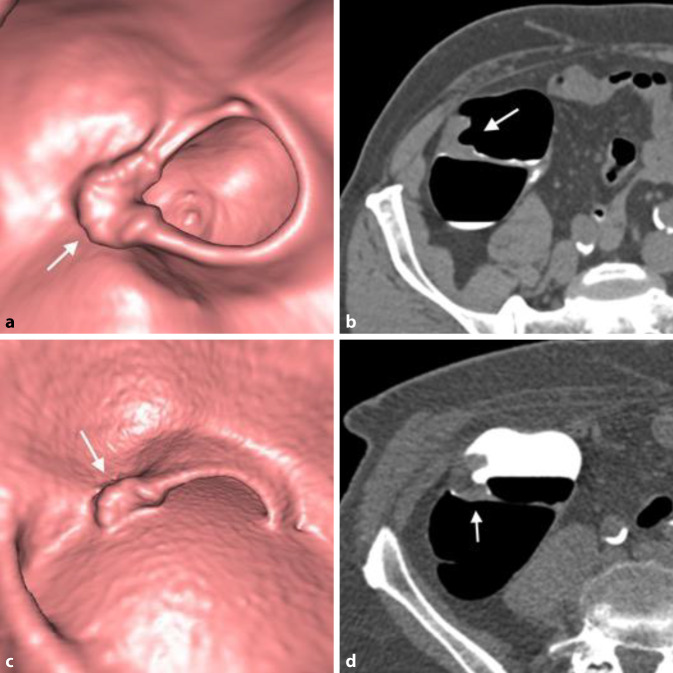

Die opportunistische Vorsorge ist individuell und erfolgt personenbezogen entweder auf Eigeninitiative oder auf Empfehlung eines Arztes. Die CTK kann als opportunistische Vorsorgeuntersuchung eingesetzt werden (Abb. 7). Teilnehmende Personen sollten allerdings, wie auch bei anderen Screening-Untersuchungen, über die spezifischen Testcharakteristika, Vorteile und Risiken informiert werden. Das betrifft besonders das selektive Polypenmanagement in Bezug auf kleine Polypen, aber auch die Notwendigkeit einer koloskopischen Intervention bei positiven Befunden. Neben der ESGE-ESGAR empfehlen auch die United States Preventive Service Task Force (USPSTF) und die American Cancer Society die CTK zur Dickdarmkrebsvorsorge [1, 31, 32].

Bei staatlich organisierten, populationsbasierten Screening-Programmen werden Personen hingegen aktiv zu Vorsorgeuntersuchungen eingeladen. Die überwiegende Mehrheit der organisierten Dickdarmkrebs-Vorsorgeprogramme basiert gemäß der Empfehlung der Europäischen Kommission primär auf Blut-Stuhl-Tests, in erster Linie dem fäkalen immunochemischen Test (FIT; [33]). In vergleichenden europäischen Studien war die CTK bei den zur Vorsorge eingeladenen PatientInnen gleich effektiv wie die Koloskopie, allerdings weniger effektiv als drei biennale Runden FIT, was durch die bekanntlich wesentlich höhere Teilnahme an Stuhltestprogrammen bedingt war [34]. Dieser Faktor ist für den Erfolg eines Vorsorgeprogramms ausschlaggebend.

Aufgrund von fehlenden Langzeitdaten zum Einfluss der CTK auf die Tumorinzidenz und -mortalität sowie auch zur Kosteneffizienz im Vergleich zu anderen Tests wird die CTK zurzeit nicht als Erstlinientest im Rahmen organisierter populationsbasierter Programme empfohlen [1].

Allerdings besteht für solche Programme eine klare Empfehlung zum Einsatz der CTK bei mittels FIT-positiv getesteten teilnehmenden Personen, bei denen die Koloskopie nicht oder nicht vollständig durchgeführt werden kann, oder bei denen die endoskopische Abklärung abgelehnt wird. Diese Vorgangsweise kommt in einigen europäischen, organisierten, populationsbasierten Vorsorgeprogrammen zum Einsatz [24, 35].

### Kontraindikationen

Die CTK ist grundsätzlich bei Patientinnen und Patienten kontraindiziert, bei denen die Untersuchung entweder schädlich sein kann oder keine zweckdienlichen, klinisch relevanten Erkenntnisse liefern kann [1, 8, 9, 36].

Das betrifft einerseits Situationen, bei denen ein besonders hohes Risiko einer Darmperforation besteht. Im Wesentlichen handelt es sich dabei um akute abdominale Zustandsbilder sowie rezent postoperative oder postinterventionelle Situationen.

Andererseits betrifft das auch Krankheitsbilder mit hohem Neoplasierisiko, bei denen histologische Untersuchungen zum Neoplasie-Ausschluss zwingend erforderlich sind, wie beispielsweise Verlaufskontrollen bei chronisch-entzündlichen Darmerkrankungen (Tab. 3).

**Table 3:** 

Spezifische Kontraindikationen	*Akute abdominale Zustandsbilder* Akute Diarrhoe Darmobstruktion Darmperforation Symptomatische abdominale Bauchwandhernie mit Kolonbeteiligung
	*Akute Darmentzündungen:* Akute Kolitis Akute Divertikulitis
	Rezente kolorektale Operationen bzw. Laparotomie
	Rezente Polypektomie oder tiefe Biopsie
Allgemeine Kontraindikationen	Fehlende Zustimmung von PatientInnen
	Bestehende oder nicht ausschließbare Schwangerschaft
Nicht geeignet für	Routinekontrollen chronisch-entzündlicher Darmerkrankungen
	Polyposis coli
	Erkrankungen des Analkanals
	Subtotale Kolektomie
	Kinder und Jugendliche

## Diskussion

Die Durchführung der CTK nach aktuellen technischen Standards ist die Grundvoraussetzung einer Untersuchung von hoher Qualität. Nur mittels technisch einwandfreier Untersuchungen lassen sich konstant gute Ergebnisse erzielen und Fehldiagnosen vermeiden. Die wichtigsten Einsatzbereiche der CTK sind die vollständige Dickdarmvisualisierung bei inkompletter Koloskopie sowie Kontraindikationen oder Ablehnung der Koloskopie. Neben diesen etablierten klinischen Indikationen wird die CTK auch zur opportunistischen Dickdarmkrebsvorsorge empfohlen. Als nichtinvasive Untersuchung ist sie eine weniger belastende Alternative zur vollständigen Dickdarmvisualisierung. Die Kenntnis der Kontraindikationen ist Vorrausetzung für den zweckdienlichen Einsatz und die Vermeidung von Komplikationen.

## Fazit für die Praxis

- Um mit der CT-Kolonographie (CTK) gute Ergebnisse erzielen zu können, ist neben der spezifischen radiologischen Expertise auch eine hochqualitative Durchführung der Untersuchung gemäß fachspezifischen Richtlinien erforderlich.
- Um das Kolon endoluminal beurteilen zu können, ist neben einer für die speziellen Anforderungen der CTK optimierten Darmvorbereitung mit „fecal tagging“ die vollständige Gasdistension des Dickdarms notwendig.
- Eine orale Kontrastmittelgabe muss bis zu 3 h vor der Untersuchung erfolgen und kann bei Bedarf auch noch vor Ort nachgeholt werden.
- Die vollständige Dickdarmvisualisierung bei inkompletter Koloskopie und Kontraindikationen oder Ablehnung der Koloskopie sind weiterhin die wichtigsten Einsatzbereiche der CTK.
- Weiters ist die CTK eine akzeptierte Untersuchungsoption zur opportunistischen Dickdarmkrebsvorsorge.

## References

[1] Spada C, Hassan C, Bellini D (2021). Imaging alternatives to colonoscopy: CT colonography and colon capsule. European Society of Gastrointestinal Endoscopy (ESGE) and European Society of Gastrointestinal and Abdominal Radiology (ESGAR) Guideline – Update 2020. Eur Radiol.

[2] Halligan S, Wooldrage K, Dadswell E (2013). Computed tomographic colonography versus barium enema for diagnosis of colorectal cancer or large polyps in symptomatic patients (SIGGAR): a multicentre randomised trial. Lancet.

[3] de Haan MC, van Gelder RE, Graser A, Bipat S, Stoker J (2011). Diagnostic value of CT-colonography as compared to colonoscopy in an asymptomatic screening population: a meta-analysis. Eur Radiol.

[4] Pickhardt PJ, Hassan C, Halligan S, Marmo R (2011). Colorectal cancer: CT colonography and colonoscopy for detection—systematic review and meta-analysis. Radiology.

[5] Lin JS, Piper MA, Perdue LA (2016). Screening for colorectal cancer: updated evidence report and systematic review for the US preventive services task force. JAMA.

[6] Tolan DJM, Rutter MD, Plumb AA (2023). CT colonography and lower gastrointestinal cancer pathways: planning for the next decade. Clin Radiol.

[7] Neri E, Halligan S, Hellstrom M (2013). The second ESGAR consensus statement on CT colonography. Eur Radiol.

[8] American College of Radiology (2019) ACR-SAR-SCBT-MR practice parameter for the performance of CT colonography in adults. American College of Radiology. https://www.acr.org/-/media/ACR/Files/Practice-Parameters/CT-Colonog.pdf?la=en. Zugegriffen: 9. Mai 2022

[9] Mang T, Schima W, Brownstone E (2011). Consensus statement of the Austrian society of radiology, the Austrian society of gastroenterology and hepatology and the Austrian society of surgery on CT colonography (virtual Colonoscopy). Rofo.

[10] Lefere P, Gryspeerdt S, Mang T (2008). CT colonography: patient preparation and examination technique. Radiologe.

[11] Sali L, Ventura L, Grazzini G (2019). Patients’ experience of screening CT colonography with reduced and full bowel preparation in a randomised trial. Eur Radiol.

[12] Lefere PA, Gryspeerdt SS, Dewyspelaere J, Baekelandt M, Van Holsbeeck BG (2002). Dietary fecal tagging as a cleansing method before CT colonography: initial results polyp detection and patient acceptance. Radiology.

[13] Pickhardt PJ, Choi JR, Hwang I (2003). Computed tomographic virtual colonoscopy to screen for colorectal neoplasia in asymptomatic adults. N Engl J Med.

[14] Johnson CD, Chen MH, Toledano AY (2008). Accuracy of CT colonography for detection of large adenomas and cancers. N Engl J Med.

[15] Regge D, Laudi C, Galatola G (2009). Diagnostic accuracy of computed tomographic colonography for the detection of advanced neoplasia in individuals at increased risk of colorectal cancer. JAMA.

[16] American College of Radiology Comittee on Drugs and Contrast Media (2023) ACR manual on contrast media. https://www.acr.org/-/media/ACR/Files/Clinical-Resources/Contrast_Media.pdf

[17] Chang KJ, Kim DH (2018). CTC technique: methods to ensure an optimal exam. Abdom Radiol.

[18] Kim DH, Hinshaw JL, Lubner MG, Munoz del Rio A, Pooler BD, Pickhardt PJ (2014). Contrast coating for the surface of flat polyps at CT colonography: a marker for detection. Eur Radiol.

[19] Stoop EM, de Haan MC, de Wijkerslooth TR (2012). Participation and yield of colonoscopy versus non-cathartic CT colonography in population-based screening for colorectal cancer: a randomised controlled trial. Lancet Oncol.

[20] British Society of Gastrointestinal and Abdominal Radiology and The Royal College of Radiologists (2021) Standards of practice for computed tomography colonography (CTC). Joint Guidance from the British Society of Gastrointestinal and Abdominal Radiology and the Royal College of Radiologists. https://www.rcr.ac.uk/publication/standards-practice-computed-tomography-colonography-ctc-joint-guidance-british-society. Zugegriffen: 25. Febr. 2023

[21] Yee J, Kumar NN, Hung RK, Akerkar GA, Kumar PR, Wall SD (2003). Comparison of supine and prone scanning separately and in combination at CT colonography. Radiology.

[22] Lambert L, Danes J, Jahoda J, Masek M, Lisy J, Ourednicek P (2015). Submilisievert ultralow-dose CT colonography using iterative reconstruction technique: a feasibility study. Acta Radiol.

[23] Andersen K, Blondin D, Beck A (2005). Assessment of two different software solutions for the evaluation of CT colonography. Rofo.

[24] Plumb AA, Halligan S, Nickerson C (2013). Use of CT colonography in the English bowel cancer screening programme. Gut.

[25] Yasumoto T, Murakami T, Yamamoto H (2006). Assessment of two 3D MDCT colonography protocols for observation of colorectal polyps. AJR Am J Roentgenol.

[26] Mang T, Graser A, Maier A, Mueller-Mang C, Bohm G, Schima W (2008). CT colonography: pathologic findings and pitfalls. Radiologe.

[27] Mang T, Peloschek P, Plank C (2007). Effect of computer-aided detection as a second reader in multidetector-row CT colonography. Eur Radiol.

[28] Dachman AH, Obuchowski NA, Hoffmeister JW (2010). Effect of computer-aided detection for CT colonography in a multireader, multicase trial. Radiology.

[29] Copel L, Sosna J, Kruskal JB, Raptopoulos V, Farrell RJ, Morrin MM (2007). CT colonography in 546 patients with incomplete colonoscopy. Radiology.

[30] Plumb AA, Ghanouni A, Rees CJ (2017). Patient experience of CT colonography and colonoscopy after fecal occult blood test in a national screening programme. Eur Radiol.

[31] Bibbins-Domingo K, Grossman DC, Curry SJ, Davidson KW, Epling JW, García FAR, Gillman MW, Harper DM, Kemper AR, Krist AH, Kurth AE, Landefeld CS, Mangione CM, Owens DK, Phillips WR, Phipps MG, Pignone MP, Siu AL, US Preventive Services Task Force (2016). Screening for Colorectal Cancer: US Preventive Services Task Force Recommendation Statement. JAMA.

[32] Levin B, Lieberman DA, McFarland B (2008). Screening and surveillance for the early detection of colorectal cancer and adenomatous polyps, 2008: a joint guideline from the American Cancer Society, the US Multi-Society Task Force on Colorectal Cancer, and the American College of Radiology. CA Cancer J. Clin..

[33] Segnan N, Patnick J, Karsa L (2010). European guidelines for quality assurance in colorectal cancer screening and diagnosis.

[34] Sali L, Ventura L, Mascalchi M, Falchini M, Mallardi B, Carozzi F, Milani S, Zappa M, Grazzini G, Mantellini P (2022). Single CT colonography versus three rounds of faecal immunochemical test for population-based screening of colorectal cancer (SAVE): a randomised controlled trial. Lancet Gastroenterol Hepatol.

[35] Lammertink MHA, Huisman JF, Bernsen MLE (2021). Implications of colonic and extra-colonic findings on CT colonography in FIT positive patients in the Dutch bowel cancer screening program. Scand J Gastroenterol.

[36] Mang T, Schima W (2013). CT colonography: a guide for clinical practice.

